# Putting students at the center: moving beyond time-variable one-size-fits-all medical education to true individualization

**DOI:** 10.2147/AMEP.S187946

**Published:** 2019-02-28

**Authors:** Debra A Schwinn, Christopher S Cooper, Jean E Robillard

**Affiliations:** 1 Department of Anesthesia, debra-schwinn@uiowa.edu; 2 Department of Pharmacology, debra-schwinn@uiowa.edu; 3 Department of Biochemistry, University of Iowa Health Care, Iowa City, IA 52242-1109, USA, debra-schwinn@uiowa.edu; 4 Department of Urology, University of Iowa Health Care, Roy J. & Lucille A. Carver College of Medicine, Iowa City, IA 5224-1101, USA; 5 Department of Pediatrics, University of Iowa Health Care, Roy J. & Lucille A. Carver College of Medicine, Iowa City, IA 52242-1101, USA

**Keywords:** medical education, curriculum, competency-based education, self-directed learning, pedagogy, medical school, innovation, learning

## Abstract

Medical education has undergone a wave of creative innovation over the last decade, with new curricular structures, pedagogy, content, and team-based approaches. Augmenting these changes, integration of clinical and scientific principles increasingly occurs across all years of training. Given success in innovation and integration, as well as recent interest and national pilots in time-variable (competency-based) education, we propose the next important step in medical education evolution is individualization.

## Innovation

Nudged by accreditation bodies, national organizations, education consortia, a new generation of education leaders, and increased recognition of the science of adult learning, medical education has undergone unprecedented innovation over the last decade.^1^,^2^ Today’s medical students mature in a digital era where they routinely explore and answer self-generated questions with information literally at their fingertips. Such students enthusiastically embrace innovative computer-based learning such as cyber/holographic anatomy to augment cadaveric dissection. The “flipped classroom” is beginning to be the new normal, with self-directed reading/assignments completed before class so that classroom time can be devoted to interactive small group learning.^3^,^4^ New academic subjects (eg, healthcare delivery science, eHealth/telehealth, quality/safety science, clinical informatics, population health) have been introduced to various degrees across the US to prepare medical students for adequate practice into the 21^st^ century.^1^ Emphasis on evidence-based informatics in guiding patient management, while discerning (and documenting) when “standard of care” may not be appropriate for a given patient, are crucial concepts for future physicians. Personal and professional wellness concepts and practice during medical school are also emphasized so that students can begin to foster sustainable engagement with their careers, while preserving family and personal vitality.

## Integration

Adding to innovative approaches, clinical and foundational science learning is also increasingly integrated across all years.^1^ For example, clinical experience often now starts in the first week of medical school, building toward longitudinal clerkships in students’ later years. Many institutions integrate foundations of bioscience and medical information along organ system, or physiologic mechanisms, in a more efficient 18-month format concentrated at the beginning of medical school. Science concepts are readdressed on clinical rotations in more formal ways than in the past, allowing re-engagement with foundational basic science principles underpinning health and disease in the most clinically relevant context possible. Core clinical rotations are more often completed earlier in medical school, giving students enhanced opportunity to explore various disciplines before determining their career specialty. These changes also allow time for inter-professional education to be transformed into longitudinal inter-professional practice opportunities with patients/families in specific local communities, ambulatory venues, and/or through health systems partnerships.

## Individualization, the next evolutionary step in medical education

We believe that given today’s robust and ongoing curricular innovation and integration, the next natural evolutionary step in medical education is individualization. Individualization is crucial in an era of ongoing major healthcare transformation (eg, exemplified by the recent entry of retail partners into healthcare).^5^ Yet, despite new knowledge needed for healthcare transformation, for more than a century medical education has focused on teaching similar material at a similar rate to all students. Although it is self-evident that individual students master material at different rates, to date logistical challenges associated with self-paced courses have limited this option in almost all medical schools. With recent technological advancements and self-directed computer-based modules to deliver some curriculum elements, as well as improved assessments of competency, some logistical challenges are being reduced; ultimately these changes offer promise of a self-paced medical education. However, with almost all residency programs routinely beginning at the same time once a year, practical flexibility of time-variable medical education remains relatively fixed (at least presently) at annual or semi-annual increments.

In addition, while a foundation of consistent medical knowledge is crucial for competent physicians, represented by the topic areas covered by the USMLE, proactive engagement with students to captivate their passions is increasingly vital. This requires deep exposure and exploration in individualized areas of medicine and/or related fields. The current generation of medical students seeks individualized opportunities to learn at a time and place of their choosing.^6^ Combining student/faculty passion with intense learning in interdisciplinary fields related to healthcare and/or biomedicine is crucial for nurturing life-long learning and developing visionary healthcare leaders of the future. Supporting this premise, a recent study suggests that spending ≥20% overall effort engaged in aspects of work that are most meaningful to an individual significantly enhances wellness and decreases physician burnout.^7^ Fortunately, since multiple schools have begun to deliver the preclinical curriculum in 18 months instead of 24 months, there is now more opportunity and flexibility to permit development of a deeply engaging and rewarding medical education experience for individual students.

In one sense, individual exploration has been available for years to a select group of medical students who have pursued research training and/or completed combining degrees (eg, MD/PhD, MD/MPH, MD/MBA); currently such training adds a year or more to standard 4-year MD education. Recent reintroduction of accelerated (time-variable) curricula (eg, 3-year MD, combined BA/MD, Education in Pediatrics Across the Continuum [EPAC], combined MD/residency programs), and even development of a new decelerated curriculum (part-time MD initiated to serve a different demographic [*e.g*. military personnel and working students]), offer their own kind of individualization.^9^,^10^ Also, the flipped classroom structure itself facilitates a faculty member’s ability to individualize questions to ensure concepts are understood by each student within small group settings. Such new curricula and teaching methods may lead to genuine competency-based medical education, ultimately providing a gold standard for achievement of pre-defined physician skills.^2^,^8^ Note, however, that the focus of these time-variable programs remains on teaching fundamental medical skills over varying time frames. We propose it is now time to view medical education from a different perspective. Given new flexibility created by ongoing innovative and integrated medical education reform, for the first time many medical students (rather than a select few) have the possibility of individualizing their education, potentially completing dual degrees, over the standard 4 years of medical school. We believe the practice of students’ self-identifying, and following, areas of intellectual curiosity/passion will serve physicians well throughout their career.

What might such individualization look like for students with different interests? Consider a medical student interested in a surgical/medicine specialty who is also committed to enhancing efficiency of patient flow through their healthcare system. Such a student might consider additional training (possibly resulting in a Master’s Degree) in implementation science (through college of engineering), quality/safety science (through college of medicine/public health), or pharmacoeconomics (through college of pharmacy), integrating additional course work and research projects across a 4-year MD. Such a dual degree-trained graduate would be uniquely suited to join teams evaluating therapeutic efficacy and cost/benefit ratios of innovative new approaches or technologies encountered during healthcare reform. Other students interested in the scientific underpinnings of medicine could explore individualized training in biomedical research (eg, clinical genetics/genomics, neuroscience, bioinformatics, physiology, biophysics, nanoscience, regenerative medicine, biomaterials, etc); such students would be ideally situated to compete for physician scientist training positions within highly competitive residency programs. Finally, consider a medical student planning on practicing rural medicine, who is also interested in informatics and telehealth. Such a student could individualize his/her curriculum by completing course work in clinical informatics (using web, classroom, and project-based education venues) and adding telehealth rotations. Ultimately this physician could become a catalyst in his/her local community, hospital, and region by developing new transformative models for patient care in vast rural regions of the country.

It is important to note that a focus on individualization does not mean that every student will choose to complete additional course work beyond basic biomedical study. Being well prepared for residency is fundamental and particularly for students who struggle academically, full-time commitment to learning clinical medicine over 4 years may be critically important for success. Other students who want to start residency early could choose an intense 3-year medical education option where available. Still others with deep outside interests unrelated to biomedicine (eg, triathlon aficionados or perhaps some men/women with young children), may decide to individualize by focusing more on work/life balance while becoming well-prepared physicians. The key is to provide flexibility and opportunity in the curriculum for individualized choice.

For those who choose to combine their life passion(s) with medical education, the critical component is ensuring that interested students have the possibility of a “deep dive” into the learning of new and innovative tools that will equip them to ask crucial questions, identify knowledgeable collaborators, and lead healthcare transformation. Rather than being “undifferentiated stem cells” (students with basic/fundamental medical skills), we posit that medical students should have the opportunity to become “differentiated,” by obtaining deeper knowledge based on their individualized passions. In so doing, they become more engaged active learners, better satisfied with their education, and uniquely able to determine their own career path, which may ultimately translate into increased innovation, long-term career satisfaction, and improved patient outcomes. Such an approach would facilitate preparation of 21^st^-century physicians with requisite knowledge and tools needed to transform healthcare and biomedical research wherever they practice.

## References

[1] American Medical Association (2017). Creating a Community of Innovation.

[2] Josiah Macy Jr. Foundation Achieving competency-based, time-variable health professions education. https://macyfoundation.org/publications/conference-summary-achieving-competency-based-time-variable-education.

[3] Schwartzstein RM, Roberts DH (2017). Saying goodbye to lectures in medical school – Paradigm shift or passing fad?. N Engl J Med.

[4] Waldrop MM (2015). Why we are teaching science wrong, and how to make it right. Nature.

[5] Cassel CK (2018). Can retail clinics transform health care?. JAMA.

[6] Evans KH, Ozdalga E, Ahuja N (2016). The medical education of generation Y. Acad Psychiatry.

[7] Shanafelt TD, West CP, Sloan JA (2009). Career fit and burnout among academic faculty. Arch Intern Med.

[8] Gruppen LD, Mangrulkar RS, Kolars JC (2012). The promise of competency-based education in the health professions for improving global health. Hum Resour Health.

[9] Stamy CD, Schwartz CC, Phillips DA, Ajjarapu AS, Ferguson KJ, Schwinn DA (2018). Time-variable medical education innovation in context. Adv Med Educ Pract.

[10] Schwartz CC, Ajjarapu AS, Stamy CD, Schwinn DA (2018). Comprehensive history of 3-year and accelerated US medical school programs: a century in review. Med Educ Online.

